# Microbiome Dynamics in a Large Artificial Seawater Aquarium

**DOI:** 10.1128/AEM.00179-18

**Published:** 2018-05-01

**Authors:** Nastassia V. Patin, Zoe A. Pratte, Matthew Regensburger, Eric Hall, Kailen Gilde, Alistair D. M. Dove, Frank J. Stewart

**Affiliations:** a School of Biological Sciences, Georgia Institute of Technology, Atlanta, Georgia, USA; b Georgia Aquarium, Atlanta, Georgia, USA; Chinese Academy of Sciences

**Keywords:** aquarium, marine microbiology, metagenomics, metatranscriptomics, microbiome

## Abstract

Artificial habitats for animals have high commercial and societal value. Microbial communities (microbiomes) in such habitats may play ecological roles similar to those in nature. However, this hypothesis remains largely untested. Georgia Aquarium's Ocean Voyager (OV) exhibit is a closed-system aquatic habitat that mimics the oligotrophic open ocean and houses thousands of large marine animals, including fish, sea turtles, and whale sharks. We present a 14-month time series characterizing the OV water column microbiome. The composition and stability of the microbiome differed from those of natural marine environments with similar chemical features. The composition shifted dramatically over the span of 2 weeks and was characterized by bloom events featuring members of two heterotrophic bacterial lineages with cosmopolitan distributions in the oceans. The relative abundances of these lineages were inversely correlated, suggesting an overlap in ecological niches. Transcript mapping to metagenome-assembled genomes (MAGs) of these taxa identified unique characteristics, including the presence and activity of genes for the synthesis and degradation of cyanophycin, an amino acid polymer linked to environmental stress and found frequently in cyanobacteria but rarely in heterotrophic bacteria. The dominant MAGs also contained and transcribed plasmid-associated sequences, suggesting a role for conjugation in adaptation to the OV environment. These findings indicate a highly dynamic microbiome despite the stability of the physical and chemical parameters of the water column. Characterizing how such fluctuations affect microbial function may inform our understanding of animal health in closed aquaculture systems.

**IMPORTANCE** Public aquariums play important societal roles, for example, by promoting science education and helping conserve biodiversity. The health of aquarium animals depends on interactions with the surrounding microbiome. However, the extent to which aquariums recreate a stable and natural microbial ecosystem is uncertain. This study describes the taxonomic composition of the water column microbiome over 14 months in a large indoor aquatic habitat, the Ocean Voyager exhibit at the Georgia Aquarium. Despite stable water column conditions, the exhibit experienced blooms in which the abundance of a single bacterial strain increased to over 65% of the community. Genome analysis indicated that the OV's dominant strains share unique adaptations, notably genes for storage polymers associated with environmental stress. These results, interpreted alongside data from natural ocean systems and another artificial seawater aquarium, suggest a highly dynamic aquarium microbiome and raise questions of how microbiome stability may affect the ecological health of the habitat.

## INTRODUCTION

Microbial communities (“microbiomes”) perform critical functions that maintain ecosystem health and stability (1–3). In the surface ocean, microbes drive the cycling of carbon, nitrogen, and other elements, such as sulfur, as well as the atmospheric exchange of these elements (4). Water column microbes also affect the microbiomes of fish and other higher organisms, both as beneficial symbionts and as agents of disease (1, 5). Saltwater aquarium systems are designed to mimic natural marine environments as closely as possible, including housing resident microbes that process nitrogenous waste and prevent pathogenic infections. Hobbyists and aquaculture professionals have long recognized that an “unhealthy” aquarium microbiome can lead to dangerous levels of ammonia or outbreaks of fish diseases (6), to the extent that there is a thriving market for bacterial additives (“probiotics”) meant to prevent these conditions (7–9). However, the long-term dynamics of large aquarium microbiomes and their degree of resemblance to those of the natural environment are largely unknown. Furthermore, while the health of aquarium megafauna, like whales and sharks, likely relies on a stable and balanced microbiome (10), animal-microbe interactions in closed aquatic systems are poorly studied. Aquaria, particularly those that do not experience water changes, therefore provide a unique opportunity to study microbiome diversity, composition, and stability in a closed saltwater environment as well as the links between the water column microbiome and animal health. Aquarium microbiomes, if sampled over time alongside chemical parameters, may also be useful systems for exploring factors driving microbial genome diversification and function.

Literature on aquarium microbiomes, particularly in large systems, is sparse. The Shedd Aquarium in Chicago, IL, recently initiated a long-term project to study the microbiomes of their saltwater, freshwater, and animal-associated microbiomes (Shedd Aquarium Microbiome Project [https://www.sheddaquarium.org/aquariummicrobiomeproject/]), an effort that will certainly contribute to the existing body of knowledge and illuminate links between habitat management, microbiome composition, and animal health. Indeed, a recent publication by Van Bonn et al. showed that water column microbiome diversity and evenness increased and stability decreased after a 90% water change in a 1,600-gallon saltwater habitat (11). That study provided evidence for the rapid turnover of microbial taxa following water changes in aquariums. However, in larger systems that use other methods to maintain water quality, the water column microbiome's stability and response to perturbations may be very different.

The Ocean Voyager (OV) exhibit at the Georgia Aquarium is the largest indoor aquatic habitat in the United States. It contains approximately 6.3 million gallons (23,814,000 liters) of artificial seawater and houses a diverse array of teleost and cartilaginous fish, including whale sharks, manta rays, and other charismatic megafauna, such as sea turtles. The water column is cycled through a series of treatments, including foam fractionation, sand filtration, sulfur-based denitrification (SDN), and ozonation. In August 2016, turbidity noticeably increased in the OV water column, suggesting a potential microbial bloom. This event occurred in the early stages of a long-term sampling project and provided an opportunity to study community dynamics leading up to and following the event, which did not adversely affect animal health but decreased visibility in the exhibit, which is viewed by 2.4 million visitors every year. Because the OV is a closed system that does not experience water changes, and chemical parameters are maintained within a narrow range, we hypothesized that the water column microbiome would be relatively stable over time, with the turbidity event being an exception.

We present a 14-month time series of OV microbiome data that suggest the opposite, highlighting surprising dynamics in the community taxonomic composition. A comparison with similar data sets from another artificial seawater aquarium (Shedd Aquarium) and three geographically distinct surface water column samples supports the finding that artificial systems are more variable and diverse than natural marine systems. Metagenomic and metatranscriptomic analyses were then performed on samples collected from two time points characterized by very different communities and used to provide insight into key taxa associated with microbiome compositional shifts. These analyses identified three nearly complete and transcriptionally active metagenome-assembled genomes (MAGs) representing bacteria generally observed at low relative abundances in the open ocean but with dominant roles and potentially unique adaptations in the OV system. We discuss the potential determinants of OV microbiome dynamics and implications for understanding aquarium microbiome ecology and diversification.

## RESULTS

### Quantitative PCR (qPCR) and turbidity data.

**FIG 1:**
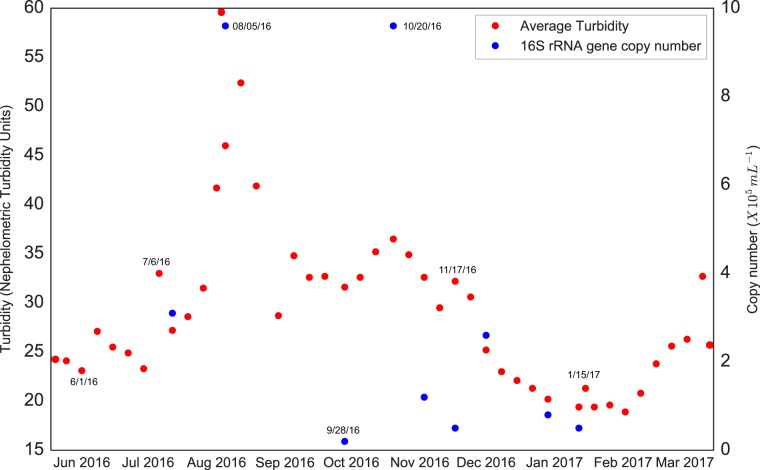
Average turbidity and 16S rRNA gene counts in the Ocean Voyager on select dates spanning the sampling time period. Key dates are labeled for clarity.

Turbidity spikes occurred during the sampling period. A primary spike occurred in August 2016, with turbidity increasing from ∼25 nephelometric turbidity units (NTUs) in the month prior to a peak of 60 NTU on 5 August (Fig. 1). Thereafter, turbidity dropped to ∼20 NTUs, before increasing to 36 NTUs during a secondary spike in late October. Bacterial 16S rRNA gene copy numbers roughly matched the turbidity trends, with numbers increasing by almost an order of magnitude to peak at 9.6 × 10^5^ copies/ml during both the primary and secondary spikes. Based on these results and the assumption that the turbidity increases were linked to microbial growth, microbiome samples were classified as bloom, nonbloom, or transition-state samples, with the latter encompassing samples collected between nonbloom and peak-bloom conditions (see Table S2 in the supplemental material).

The measured water column chemical and physical parameters varied minimally over the course of the 14-month sampling period (Table S1). The parameters with the highest correlations (*R*^2^) with turbidity values and gene copy numbers were dissolved oxygen (*R*^2^ values of 0.412 for turbidity and 0.261 for copy numbers) and nitrate (*R*^2^ values of 0.308 for turbidity and 0.371 for copy numbers) levels, with all other correlations being much lower or inconsistent between turbidity and copy numbers (Table S1).

### Amplicon time series.

**FIG 2:**
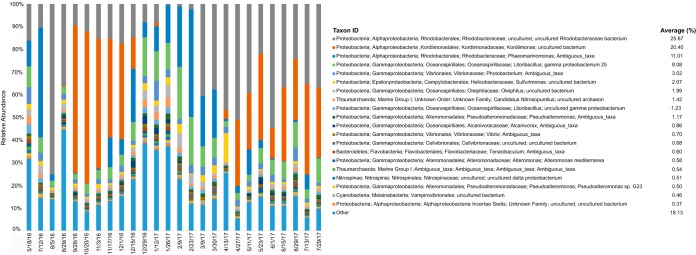
Taxonomic composition of the Ocean Voyager microbiome over time. The top 20 most abundant amplicon sequence variants (ASVs) are labeled with different colors, and all other taxa are grouped together at the bottom of each bar as “Other.” The average relative abundance of each ASV over all samples is provided next to the key.

The community composition in the OV was dominated at all times by one of three alphaproteobacterial taxa: one amplicon sequence variant (ASV) classified only at the family level (Rhodobacteraceae), one of the genus Phaeomarinomonas (also family Rhodobacteraceae), and one belonging to the genus Kordiimonas (family Kordiimonadaceae). At 23 of 26 time points, the combined relative abundances of these taxa exceeded 25%. At 19 of 26 time points, they exceeded 50%. The community of the first bloom event (August) was dominated by the two Rhodobacteraceae ASVs, which comprised 75% of the community, at approximately 724,000 16S rRNA gene copies/ml, assuming uniform 16S gene copy numbers among taxa (Fig. 2). The second bloom event was dominated by the Kordiimonas sp. ASV, which sustained over 40% relative abundances between September and December 2016 (Fig. 2), reaching a peak of 67% of the community on 20 October 2016 at ∼645,000 16S rRNA copies/ml.

**FIG 3:**
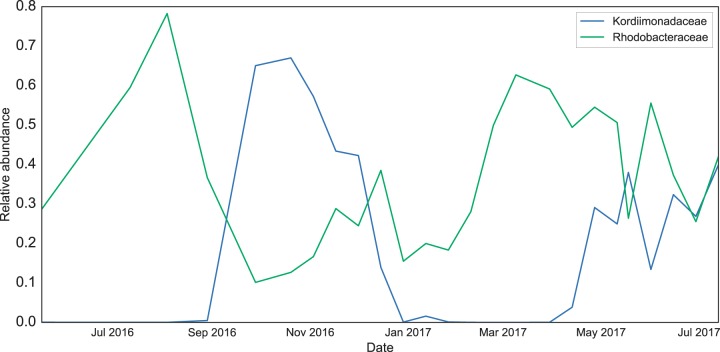
Relative abundances over time of the Rhodobacteraceae and Kordiimonadaceae families, respectively. Each family is represented by multiple ASVs, including the three dominant ASVs discussed in the text.

The community experienced a rapid switch from one dominant taxon to another between the August and October blooms, after which the relative abundances of all three dominant taxa declined. The abundances of the two Rhodobacteraceae ASVs then steadily increased between December 2016 and April 2017, when the Kordiimonas ASV rebounded to ∼30% of the community. Indeed, the relative abundances of the Rhodobacteraceae and Kordiimonadaceae families (all associated ASVs included) were inversely related throughout the sampling period (Fig. 3). These two families oscillated most dramatically over the first 9 months of the time series but exhibited more-equal representation between April and July 2017 (Fig. 2 and 3).

**FIG 4:**
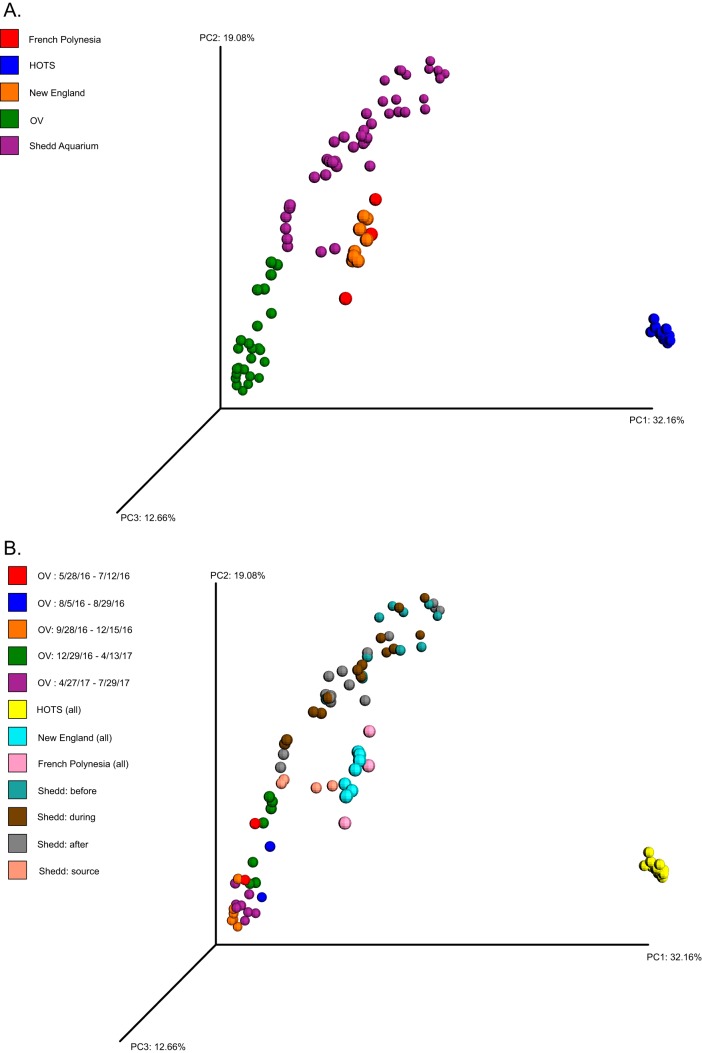
Principal-component analysis comparing microbiome compositions among samples from the Ocean Voyager and the Shedd Aquarium (aquarium water samples) as well as Station ALOHA (HOTS) (25 m), French Polynesia (<1 m), and New England (<1 m) (natural seawater samples). Clustering is based on the weighted UniFrac metric generated from the 16S rRNA gene amplicon data of all samples. (A) Samples are colored by environment. (B) The aquarium samples are further classified by collection time period (OV) or, for the Shedd Aquarium data, by experiment stage (before, during, and after a 90% water change; the data set also contains a sample of the replacement water used for the water change). HOTS, French Polynesia, and New England samples are colored based only on environment, as in panel A.

Comparison of the OV data with data from surface water samples from the Hawai’i Ocean Time-Series (HOTS), French Polynesia, New England, and the Shedd Aquarium water column showed that the aquarium data sets exhibit higher intersample variability in composition (beta diversity) than natural ocean communities (Fig. 4). OV communities varied substantially over the 14-month sampling period, as did the Shedd Aquarium communities, whereas communities from French Polynesia, HOTS, and New England clustered more tightly in multivariate space despite the fact that the natural ocean data sets spanned seasons (HOTS and New England data) (Fig. 4). The tropical samples exhibited significantly lower alpha diversity than did the New England and aquarium samples (Fig. S2) (Shannon’s diversity index [H’] *P* <<< 0.001; Simpson's evenness index [E] *P* < 0.001). However, the HOTS communities were notably distinct from other surface water and aquarium samples due largely to their high relative abundances of cyanobacteria and the complete absence of members of the Archaea (Fig. 4 and Fig. S2). This discrepancy is likely due in part to the different primer sets used to generate the HOTS data (23F and 534R, targeting the V1-V3 region of bacterial small-subunit [SSU] rRNA genes) compared to those used for all other samples (12).

The dominant phylum in the OV communities at all time points was Proteobacteria, which averaged near 90% over all time points (Fig. S3). In contrast, the abundances of cyanobacteria averaged just 1.1% over all time points and never reached abundances of >3.5%. Other natural marine communities were also dominated by Proteobacteria, although some samples featured high relative abundances of Bacteroidetes (French Polynesia and New England [fall samples]) and Cyanobacteria (French Polynesia) compared to those of the OV communities. The Shedd Aquarium exhibited high relative abundances of Firmicutes, particularly anaerobic taxa of the order Clostridia. Despite differences in alpha and beta diversity values, the OV and Shedd Aquarium microbiomes consistently contained certain groups observed commonly in natural marine microbial communities, notably gammaproteobacteria of the order Oceanospirillales and members of the Thaumarchaeota (Fig. 2 and Fig. S3).

### Metagenome-assembled genomes.

Two coassemblies were generated: one with the 5 August 2016 sample (hereafter “0805”) and one with the 28 September 2016 sample (hereafter “0928”). Assembly statistics for both coassemblies (minimum contig lengths of 1,000 and 2,500 bp, respectively), as determined by MetaQUAST (13), are given in Table S3 in the supplemental material. The longest contig in both coassemblies was 1,507,463 bp. *N*_50_ values were 2,581 and 90,952 bp for the 1,000-bp and 2,500-bp coassemblies, respectively. Sample 0805 reads produced 10 MAGs, 3 of which were nearly complete (>97.7%) when mapped to the coassembled metagenomes (Table S5), while the sample 0928 reads generated 6 MAGs, 3 of which were nearly complete (>96.8%) (Table S5). Four eukaryotic rRNA sequences were identified, and the associated contigs were removed from the MAGs with anvi'o (Table S8). Bacterial rRNA genes were detected in only 5 of the 16 total MAGs, and in one case (MAG 0928_002), they were likely spuriously binned, as judged by comparison to the MAG taxonomic classification based on protein-coding sequences (see below); contigs containing these sequences (partial 16S and 23S rRNAs) were removed from the analysis (Table S8).

**FIG 5:**
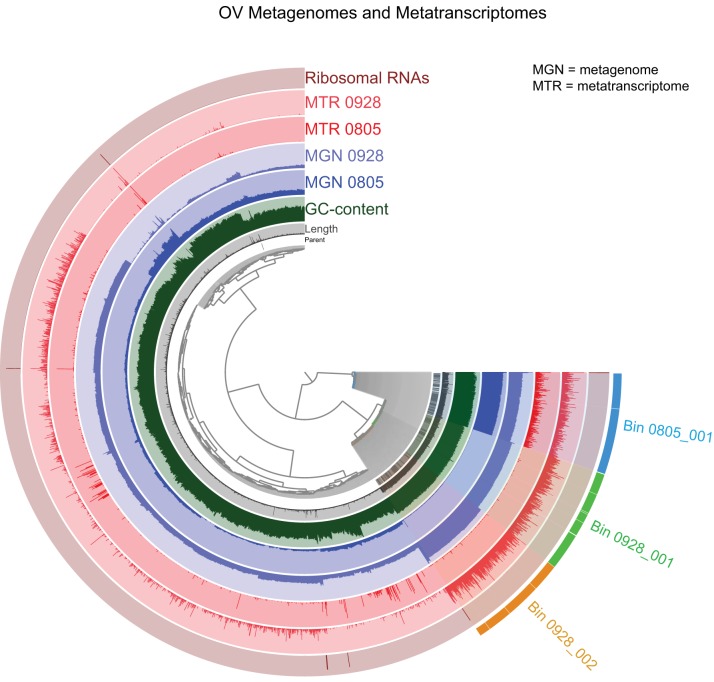
Distribution and coverage of reads from each metagenome and metatranscriptome mapped onto the metagenome coassembly, visualized in anvi'o. Each branch of the dendrogram represents a contig or a contig split, with splits from the same contig denoted by the “parent” layer. Branches are clustered by k-mer composition and contig, or split, with length being represented in the “length” layer. GC content as a percentage is provided in the third layer. The two blue layers represent the two metagenome samples, while the two red layers represent the metatranscriptomes. The presence of rRNAs is shown in the outer layer. The coverage for each sample is shown in its respective circle. The three alphaproteobacterial MAGs used for phylogenetic analysis and transcriptome comparisons are shown in different colors on the bottom right part of the dendrogram.

**FIG 6:**
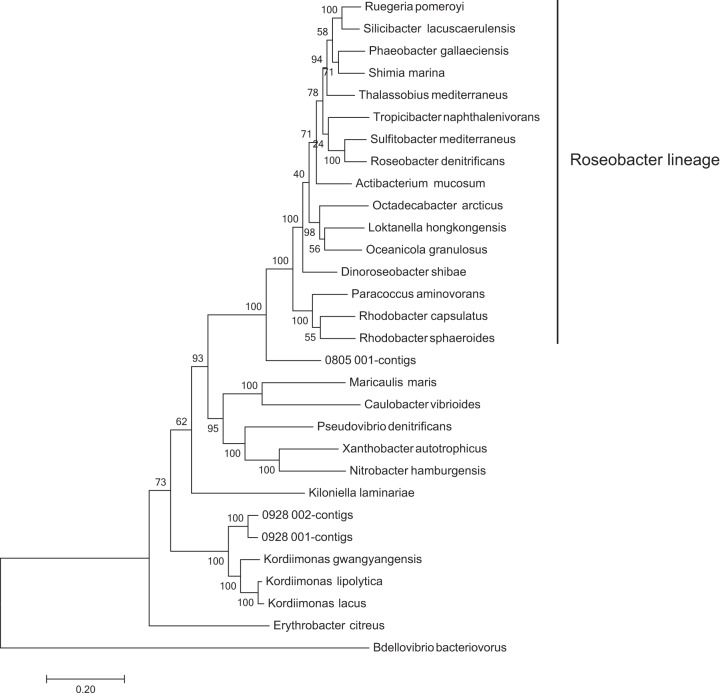
Phylogenetic tree showing the placement of three near-complete MAGs recovered from the Ocean Voyager relative to genomes of diverse marine alphaproteobacteria. The phylogeny is based on an amino acid alignment of 43 concatenated single-copy marker genes. Taxa belonging to the Roseobacter clade, as described previously (55), are marked for clarity.

The generation of bins using reads from each of the two samples individually showed that three of the MAGs were present in both samples (Table S5). For subsequent analyses, we used the representative MAGs with the highest completeness: MAGs 0805_001, 0928_001, and 0928_002 (Fig. 5). Over 60% of the sample 0805 reads recruited to MAG 0805_001 (Table S6). Of the sample 0928 reads, over 40% recruited to MAG 0928_001, and just over 12% recruited to MAG 0928_002 (Table S6). These MAGs did not contain confidently assigned 16S rRNA genes that could be compared to the amplicon time series data. However, phylogenetic analysis of single-copy protein-coding genes placed MAGs 0928_001 and 0928_002 in a highly supported clade of the genus Kordiimonas (Fig. 6) and placed MAG 0805_001 in a highly supported clade with diverse genera of the Rhodobacteraceae. These results are consistent with the high abundances of unclassified Kordiimonas and Rhodobacteraceae in the amplicon data (Fig. 2), suggesting that these MAGs likely represent relatives of the ASVs dominant at the time of collection.

Functional annotation of the MAGs showed that all three MAGs contain genes for enzymes associated with aerobic respiration and heterotrophic metabolism, including key genes for the tricarboxylic acid (TCA) cycle and glycolysis (Table S9). All identified genes that were also transcribed are presented in Table S8 in the supplemental material. There was no evidence of genes related to anaerobic respiration, photosynthesis, or other forms of autotrophy. Several genes related to tolerance to oxidative stress were identified, including superoxide dismutases and peroxidases (Table S10).

**FIG 7:**
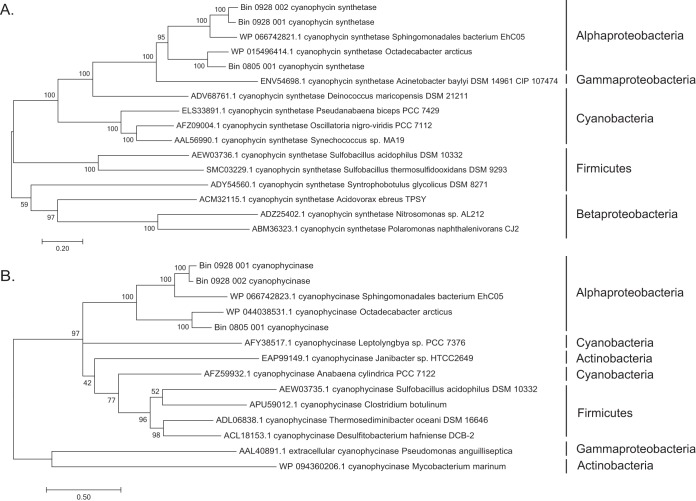
Phylogenetic trees showing the placement of the cyanophycin synthetase (CphA) (A) and cyanophycinase (CphB) (B) amino acid sequences recovered from each of the three alphaproteobacterial MAGs. Homologs included in the phylogenies include those from other Alphaproteobacteria as well as Gammaproteobacteria, Betaproteobacteria, Cyanobacteria, Firmicutes, and Clostridia.

All three MAGS contain genes required for the biosynthesis (*cphA*, encoding cyanophycin synthetase) and degradation (*cphB*, encoding cyanophycinase) of cyanophycin, an amino acid polymer found commonly in cyanobacteria but rarely in heterotrophic bacteria (14, 15). In all MAGs, the genes for CphA and CphB are located adjacent to each other on the same contig. These genes cluster phylogenetically with homologs from other proteobacteria and separately from those of Cyanobacteria, Firmicutes, and Actinobacteria (Fig. 7).

Putative plasmids were detected throughout the metagenome coassemblies, although most of them could not be assigned definitively to a MAG. These plasmids range in size from 10 to 30 kb and contain genes related to conjugation and toxin production, including those encoding integrases, the toxins ParE1 and HigA2/HigB2, type IV secretion system proteins, and the conjugal transfer protein TraG (identified in all putative plasmids) (Table S11). Only one of the putative plasmids was binned into one of the three high-quality MAGs, MAG 0928_002 (Table S11).

### Metatranscriptomes.

Approximately 62% and 77% of the August 2016 (sample “0805”) and September 2016 (sample “0928”) transcripts, respectively, could be used for functional analysis after the merging and removal of the remaining rRNAs (see Table S4 in the supplemental material). Of the processed reads, 10.5% from sample 0805 mapped to the coassembled metagenome (>1,000 bp), compared to 67.9% from sample 0928. Among the reads that mapped to the coassembly, most were recruited to the same MAGs that dominated the metagenome (Fig. 5 and Table S6).

The taxonomic compositions of the protein-coding transcripts varied substantially between time points. Bacterium-associated sequences comprised 68% of the data for sample 0805 but 91% for sample 0928. This proportional shift was driven primarily by an enrichment of eukaryote-associated transcripts in sample 0805, to 31% of the total taxonomically identifiable transcripts, compared to 6% in sample 0928 (Table S4). Of these transcripts, the majority were classified across a broad range of taxonomic groups, making it challenging to associate a single eukaryotic group to the August bloom. The same trends were not observed for the rRNA-depleted metagenomic reads. Over 97% of the metagenomes of both samples 0805 and 0928 were classified as bacterial reads, with 1% or fewer being assigned to archaea or eukaryotes (Table S4).

A large proportion (∼10%) of the sample 0805 bacterial transcripts were affiliated with the genus Sulfurimonas, relative to ∼0.001% of the sample 0928 bacterial transcripts. Sulfurimonas-like transcripts in this sample were more abundant than those affiliated with the entire alphaproteobacterial subphylum, which made up about two-thirds of the sample 0928 transcripts (Table S4). Sample 0805 also contained more eukaryotic and fewer archaeal reads than did sample 0928.

Counts of transcripts encoding CphA and CphB, combined, were 60 and 118 for samples 0805 and 0928, respectively (per 3,340,400 transcripts analyzed per sample) (Table S12). Of these transcripts, CphB transcripts were 25 times more abundant in sample 0928 than in sample 0805 and were affiliated (best BLAST hit) with cyanophycinases from 14 taxa (Table S12).

## DISCUSSION

This study assessed the stability of the pelagic microbiome over time in a large closed-system aquatic habitat, the Ocean Voyager exhibit at the Georgia Aquarium. The sampling period encompassed fluctuations in OV water clarity, including primary and secondary turbidity peaks in August and October 2016. These turbidity events coincided with spikes in bacterial 16S rRNA gene counts. However, the relationship between turbidity and gene counts was not constant; compared to the August event, the October event involved a modest turbidity increase but a large increase in gene counts (Fig. 1). This anomaly could be explained by changes in microbiome composition between turbidity events, potentially affecting the average cell size or number of rRNA operons per cell. Indeed, the composition of the OV microbiome shifted markedly from August to October. During the August event, two ASVs in the alphaproteobacterial family Rhodobacteraceae comprised 75% of the 16S rRNA gene sequences (uncultured Rhodobacter sp. and Phaeomarinomonas sp.) (Fig. 2). By late October, a single ASV of the alphaproteobacterial genus Kordiimonas comprised 67% of sequences. This ASV had already become dominant (>60%) 1 month prior to the October turbidity maximum and also occurred at a high abundance (>30%) under less-turbid conditions in 2017 (Fig. 1 and 2). In contrast, the Rhodobacteraceae ASVs more closely tracked with turbidity, increasing in representation prior to the August turbidity peak and again during a turbidity increase in March 2017 (Fig. 1 and 2). These patterns suggest that some, but not all, of the dominant taxa are indicators of OV turbidity events and that some events may involve increases in the abundances of multiple members of the community. The relationship between turbidity and bacterial gene counts could also vary if turbidity was influenced by other factors, such as growth by microeukaryotes. This possibility is supported by an enrichment of eukaryotic sequences in metatranscriptome data in August relative to October, although absolute counts of microeukaryotes were not determined, and the metagenome taxonomic assignments do not support a eukaryotic enrichment. Overall, the time series data confirm a potential for large changes in the microbiome composition and abundance (blooms) and suggest that such changes are associated with declining water clarity.

The observed fluctuations in taxonomic composition, over periods as short as 2 weeks (Fig. 2), underscore the highly dynamic nature of the OV microbiome. Similarly high temporal variability is also evident for the much smaller exhibit represented by the Shedd Aquarium data set (Fig. 4) (11). These temporal changes were significantly greater (higher beta diversity) than those in natural oligotrophic marine environments, including Station ALOHA (HOTS; 25-m depth) and surface waters in French Polynesia and New England (Fig. 4), despite the potential for community change in natural systems due to winds, storms, and seasonality in light and temperature (12). Indeed, the observed OV microbiome shifts occurred despite relatively stable chemical and physical conditions (see Table S1 in the supplemental material). This temporal variability suggests that the OV microbiome responded to changes in water conditions that occurred below our detection limits or to unmeasured factors, potentially including the episodic release of microbes from other reservoirs in the system (e.g., filtration systems). The OV's intense ozone treatment may also affect compositional dynamics. Bacterial cell numbers in the OV (inferred from 16S rRNA gene counts) are low compared to those in natural environments (1 × 10^5^ to 2 × 10^5^ copies/ml under nonturbid conditions versus 5 × 10^5^ to 7 × 10^5^ copies/ml at Station ALOHA [16]), presumably due partly to cell death from ozonation. At such low abundances, the community may be particularly susceptible to compositional changes associated with population bottlenecks, for example, due to increases in ozone intensity to combat turbidity. Another possible explanation for the spike in turbidity is a physiological response of the dominant taxa to ozonation by forming cell aggregates. Some marine bacteria, including those of the Roseobacter clade, have adaptations for surface colonization that can be induced by environmental factors such as oxidative stress (17). However, we found little evidence to support this hypothesis. An examination of water samples under a compound microscope showed a homogeneous distribution of microbial cells (data not shown), and extensive analysis of the MAGs revealed no genes related to biofilm formation and surface colonization. The mechanisms underlying the changes in the OV microbiome remain cryptic but likely involve a range of factors, some of which may be stochastic and challenging to detect and control.

Despite facing artificial conditions and potentially intense selection pressures (e.g., ozonation), the alpha diversity of the OV and Shedd Aquarium microbiomes is much higher than that of surface water communities in Hawaii and French Polynesia and comparable to that of temperate coastal communities from New England (Fig. S2). This diversity is perhaps surprising given an apparent dearth of water column niches due to a lack of vertical stratification and the absence of a well-developed phototroph niche. The relative abundance of cyanobacteria in the aquariums, for example, is notably lower than that in all ocean environments (Fig. S3), which is consistent with the limited lighting over the OV. Furthermore, the New England water experiences large seasonal variations in physical parameters such as temperature (18), unlike the OV, where the temperature is maintained within a very narrow range (Table S1). One possible explanation for the maintenance of high OV microbiome diversity is seeding of the water column from filtration systems or the aquarium's animal inhabitants. Such sources may include microbes from both oxic and anoxic biofilms in the sand and sulfur-based denitrification filters or on the surface or in the guts of fish. Evidence of carryover from other reservoirs is seen in the high proportion of sample 0805 metatranscriptome sequences matching Sulfurimonas, a genus of sulfur-oxidizing epsilonproteobacteria that is abundant in sulfur-based denitrification filters (our unpublished data). A Sulfurimonas species ASV was among the 10 taxa with the highest relative abundances (Fig. 2), averaging about 2% of the water column community. This pattern supports the idea that sulfur-oxidizing microbes are entering the water column from the filtration system. Furthermore, given that the exhibit was not established by using natural seawater, it is assumed that the majority of microbial taxa enter the OV attached to food or live animals. Indeed, several microbial groups prevalent in the OV samples, notably members of the Oceanospirillales and marine Vibrionaceae, are commonly found in association with diverse marine fish and invertebrates (19–21). The high density of OV animal inhabitants, which include some of the largest fish species in the ocean and are fed daily from external sources, may result in the pelagic OV microbiome being disproportionately represented by taxa more typical of host-associated niches.

Although the richness is high in the OV and potentially impacted by the episodic input of taxa from other reservoirs, certain microbes are pervasive across time and play dominant roles in water column community dynamics. In all but 6 samples, at least one of the three dominant alphaproteobacterial ASVs (Rhodobacteraceae and Kordiimonas) comprised >50% of the community. When the Kordiimonas ASV was present, its abundance correlated inversely with that of the two Rhodobacteraceae ASVs combined (*R*^2^ = 0.69) (Fig. S4), although these oscillations subsided in the last 3 months of the time series, when these groups were detected at similarly high levels (Fig. 3). The dominance of these taxa suggests the potential for niche overlap as well as important ecological roles in the microbiome.

To explore the function and adaptations of these dominant OV taxa, we analyzed three near-complete MAGs recovered from shotgun metagenome data sets (Fig. 5). MAG 0805_001 dominated the August bloom (recruiting 61.8% of metagenome reads and 88.7% of those that mapped to the coassembly) and is closely related to the Roseobacter clade of the alphaproteobacterial Rhodobacteraceae (Fig. 6), which agrees with the taxonomic assignment of the dominant 16S rRNA amplicon from the same sample. The Rhodobacteraceae are also among the most pervasive groups of marine microbes (22). Roseobacters in particular have been recovered from most marine ecological niches and play dominant roles in chemical cycles as heterotrophs, although diverse physiologies, including methylotrophy, photoheterotrophy, and mixotrophy, have been described for this group (23, 24). MAG 0805_001 did not contain genes for CO_2_ fixation, anoxygenic photosynthesis, or alternative respiration strategies but contained and transcribed genes for the TCA cycle, glycolysis, and the aerobic respiratory electron transport chain (Table S9), suggesting that this organism is likely aerobic and heterotrophic. Furthermore, the prevalence of genes involved in oxidative stress tolerance may be linked to the regular ozonation of the OV water column, which likely enriches for microbes that are better equipped to survive high levels of radical oxygen species.

The other two MAGs (MAGs 0928_001 and 0928_002) dominated the secondary bloom in October and are closely related to each other within the alphaproteobacterial genus Kordiimonas (Fig. 6). While we recovered two Kordiimonas MAGs clearly differentiated by GC:coverage ratios, the community amplicon data for sample 0928 show only one Kordiimonas ASV (Fig. 5). Although we were not able to recover 16S rRNA genes from these MAGs for comparison, it is possible that both MAGs share identical 16S rRNA V4 sequences. This would suggest that this genus contains as-yet-unknown microdiversity, possibly related to its ability to adapt to a variety of marine environments. Indeed, Kordiimonas spp. have been isolated from a diverse range of marine environments, including sediments, ballast water, and coastal deep-sea and open-ocean water columns (25–27). They have also been observed at high relative abundances in small experimental aquaria following the introduction of macroalgae (28). The gene content and transcriptional activity of the Kordiimonas MAGs suggest that these taxa are aerobic heterotrophs, similar to MAG 0805_001 (Table S9).

The sustained dominance of the taxonomic groups represented by the MAGs suggests that these taxa may have adaptations to the unique OV environment. Interestingly, all three MAGs contain genes for synthesizing and degrading cyanophycin, an amino acid polymer thought to function as a nitrogen and carbon storage molecule during periods of environmental stress (14). Cyanophycin is synthesized nonribosomally by a synthetase (CphA) and degraded by cyanophycinases, which can be intracellular (CphB) or extracellular (CphE) (15). All three MAGs contained genes for CphA and CphB. Orthologs are found in most cyanobacteria as well as in a small but diverse group of heterotrophic bacteria (15, 29, 30). However, none of the publicly available Kordiimonas species genome sequences contain cyanophycin-related genes. This suggests that CphA and CphB of the Kordiimonas MAG, which cluster with homologs from other alphaproteobacteria (Fig. 7), were acquired horizontally. It is unclear why cyanophycin persists in the OV populations and might provide an advantage in this system. This molecule could confer protection from ozonation by an as-yet-unknown mechanism or may indicate nutrient limitation or the presence of other physiological stressors under which nitrogen and carbon storage would provide a fitness benefit. Transcript levels of *cphA* and *cphB* were low (Table S12), suggesting that cyanophycin was not produced in large amounts at the sampling times. Reliance on the biopolymer may instead be intermittent, perhaps allowing ecological versatility over fluctuating environmental or microbiome states (e.g., changes in the ozonation intensity or competitive pressure due to a shifting microbiome composition). Determining the fitness advantage provided by cyanophycin in the OV may shed light on the ecological importance of this unusual biopolymer in other systems.

The versatility of certain OV microbes is likely supported in part by gene exchange. Indeed, the meta-omic analysis identified five putative plasmids carrying and transcribing diverse genes associated with genome mobilization (Table S11 and Fig. S1). These genes included the gene encoding the conjugal transfer protein TraG, found in all five plasmids. The recovery of plasmids suggests that OV microbes may use conjugal DNA transfer to adapt to changes in water column conditions. Such genetic plasticity could also allow for rapid changes in the dominance of the community, particularly if the “seed” populations after ozonation are small.

### Conclusions.

Despite being tightly controlled, the Ocean Voyager contains a species-rich and highly dynamic microbial community subject to large fluctuations in composition. Despite high richness, the community is almost always dominated by a small number of microbial strains representing cosmopolitan marine microbial lineages. These strains, by virtue of their abundance, likely share similar niches and make proportionately large contributions to chemical turnover by the microbiome. These strains may also be models for identifying adaptations to unique OV selection pressures, which include intense environmental filtering by ozonation and potentially episodic bursts of resources and new microbial competitors from animal hosts. Investment in carbon and nutrient storage may be one such adaptation, providing excess reserves to help weather periods of environmental stress or a disruption of other physiological functions. The OV and other closed aquatic systems therefore provide opportunities for studies on the evolution of “marine” microbial taxa. Such adaptation may be facilitated by genetic exchange via plasmids and potentially by high connectivity to other reservoirs of microbial cells and genes in the OV system. The growing interest in microbiomes of animal habitats offers a chance to explore basic questions of microbial natural history, ecology, and evolution, as well as to better understand the role of microorganisms in maintaining healthy captive animals.

## MATERIALS AND METHODS

### Exhibit setup and management.

Georgia Aquarium's Ocean Voyager is an entirely closed system of 6.3 million gallons of artificial seawater (Atlanta tap water mixed with instant ocean sea salt [Instant Ocean Spectrum Products, Blacksburg, VA, USA]) designed to house primarily pelagic fauna of the open ocean. Exhibit water circulates through a filtration system employing foam fractionators (protein skimmers), sand filters, ozone contact towers, countercurrent heat exchangers, sulfur-based denitrification (SDN) vessels, and a deaeration tower (DAT).

Water from the exhibit is brought to a sump via surface skimming and bottom return lines (approximately a 2:1 ratio of surfacing skimming to bottom return). From the sump, 100% of the water is foam fractionated through protein skimmers to remove small organic compounds and then passed through sand filters to remove particulates and support the microbial nitrification of ammonia to nitrate. These filters have a gravel base and 0.45- to 0.55-mm silica filter sand designed to catch particulates such as fish waste or uneaten food. Following the sand filters, water is diverted via different side-stream processes. Countercurrent heat exchangers made of titanium plates maintain water temperature. Approximately 0.15% of the exhibit water is diverted to the SDN vessels, which are designed to promote nitrate removal by microbial autotrophic denitrification by sulfur-oxidizing bacteria. The SDN side loop is comprised of two sets of four vessels, each of which contains gravel, sulfur pellets, aragonite, and associated denitrifying microbes, primarily of the genera Thiobacillus and Sulfurimonas (our unpublished data). Two process loops provide the optimal hydraulic retention time of water in the SDN system. The first loop is pumped and recirculated continuously through the SDN vessels, while the second loop titrates a small amount of water from the exhibit into the first loop and back out again to the exhibit. All of the water from this side stream, along with 25% of the original post-sand-filter flow, also flows through an ozone contact tower, which uses ozonation to kill microbes by oxidation and to raise water dissolved oxygen levels, which are lowered by passage through the SDN system. Ozone dosing is regulated by oxidation/reduction potential (ORP) readings, with a target level of 675 mV. However, the ozonation intensity is increased intermittently to help regulate changes in water clarity. The remainder of the flow from the sand filters goes into the DAT, which also receives 100% of all side-stream processes. The DAT is filled with Brentwood medium to create a large surface area that promotes gas exchange, thereby allowing dissolved gasses, such as CO_2_ and O_2_, to reach equilibrium with the ambient environment. Water enters the DAT through spray bars at the top and trickles down through the medium. Water leaves the DAT via gravity and a series of pipes that spread flow around the exhibit.

The system filter rate is close to 130,000 gallons (491,400 liters) per min (GPM), which occurs through two “in-series” filtration loops, each of which provides a filter rate of 65,000 GPM. This process turns over the exhibit “tank-only” volume of 4 million gallons approximately once per hour. Chemical parameters within the exhibit are closely monitored and maintained with minimal variation around target levels (see Table S1 in the supplemental material). To compensate for pH shifts outside the acceptable range, sodium carbonate or sodium bicarbonate is occasionally titrated into the OV water at the sump, before filtration. The system is lit by overhead metal halide lighting and natural sunlight from a skylight over a small portion of the southern end of the exhibit.

### Metadata collection.

Water column pH and salinity were measured at Georgia Aquarium by using an Orion VersaStar meter with a conductivity cell and a Ross Ultra pH/ATC triode probe. ORP was measured using a GF Signet 2757 in-line probe. The dissolved oxygen concentration was measured *in situ* by using a HQ40d portable multimeter with a luminescent probe (Hach Company, Loveland, CO, USA). The nitrate concentration was measured by using a Dionex ICS 5000^+^ ion chromatography system with a Dionex IonPac AS23 analytical column. The nitrite concentration was measured on a DR6000 spectrophotometer (Hach) by using the U.S. EPA diazotization method. Alkalinity was determined through titration using 0.01 N HCl. All measurements and samples were taken between 06:30 and 07:30. Samples were taken directly from the habitat in a consistent location and analyzed within 5 h.

Turbidity readings were taken every 5 min daily with a Filtertrak 660 sc laser nephelometer sensor (Hach). Water for turbidity readings was pulled in from a pipe on the side of the exhibit, such that readings were taken from the middle of the water column. Values for select dates were determined by the mean of all measurements on that date. Samples were classified into three bloom condition categories based on nephelometric turbidity units (NTU), as follows: nonbloom (<30 NTU), transition (30 to 35 NTU), and bloom (>35 NTU) (see Table S2 in the supplemental material).

### Sampling scheme.

Samples for microbiome analysis were taken approximately every 2 weeks from May 2016 until July 2017 (see Table S2 in the supplemental material). At each time point, 2 liters of Ocean Voyager surface water was filtered through each of four Sterivex cartridges (0.2-μm pore size) by using a peristaltic pump. Cartridges were filled with nucleic acid stabilizing buffer (25 mM sodium citrate, 10 mM EDTA, 5.3 M ammonium sulfate [pH 5.2]), sealed, and placed on dry ice for transport back to Georgia Tech, where they were stored at −80°C until nucleic acid extraction.

### DNA extraction.

DNA was extracted from one of the four replicate Sterivex cartridges by using a custom protocol described previously (31). Briefly, cells were lysed by flushing out nucleic acid stabilizing buffer and replacing it with lysis buffer (50 mM Tris-HCl, 40 mM EDTA, 0.73 M sucrose) and lysozyme (2 mg in 40 μl of lysis buffer per cartridge) and then incubating cartridges for 45 min at 37°C. Proteinase K was added, and cartridges were resealed and incubated for 2 h at 55°C. The lysate was removed, and the DNA was extracted once with phenol-chloroform-isoamyl alcohol (25:24:1) and once with chloroform-isoamyl alcohol (24:1). Finally, DNA was concentrated by spin dialysis using Ultra-4 centrifugal filters (100 kDa; Amicon).

### Quantitative PCR assay.

qPCR was used to count total bacterial 16S rRNA gene copies in DNA extracted from a subset of samples. Total gene copy numbers were obtained by using SYBR green-based qPCR and universal bacterial 16S rRNA gene primers 1055f and 1392r, as described previously (32). Tenfold serial dilutions of DNA from a plasmid carrying a single copy of the 16S rRNA gene (from Dehalococcoides mccartyi) were included on each qPCR plate and used to generate standard curves. Assays were run with a 7500 Fast PCR system and a StepOnePlus real-time PCR system (Applied Biosystems). All samples were run in triplicate (20 μl each) and included 1× SYBR green supermix (Bio-Rad), 300 nM primers, and 2 μl of template DNA (diluted 1:100). Thermal cycling involved incubation at 50°C for 2 min to activate uracil-*N*-glycosylase (UNG), followed by incubation 95°C for 10 min to inactivate UNG, denature template DNA, and activate the polymerase and 40 cycles of denaturation at 95°C (15 s) and annealing at 60°C (1 min).

### Correlation analyses.

Turbidity values and qPCR results for 16S rRNA gene copy numbers were plotted together by using iPython and Matplotlib (33). Both time series were also tested for correlation with each of the available chemical parameters. Significance was determined by using the *R*^2^ correlation coefficient in Microsoft Excel.

### 16S rRNA gene amplicon library preparation.

Illumina MiSeq libraries were prepared by amplifying the V4 region of the 16S rRNA gene from environmental DNA, as described previously (34). Briefly, amplicons were generated by using Platinum PCR SuperMix (Life Technologies) with primers F515 and R806, encompassing the V4 region of the 16S rRNA gene (35). These primers are used primarily for bacterial 16S rRNA gene analysis but also amplify many, although not all, archaeal sequences (36). Both forward and reverse primers were barcoded and appended with Illumina-specific adapters, as described previously (37). Thermal cycling involved denaturation at 94°C (3 min); 30 cycles of denaturation at 94°C (45 s), primer annealing at 55°C (45 s), and primer extension at 72°C (90 s); and extension at 72°C for 10 min. Amplicons were analyzed by gel electrophoresis to verify the size (∼400 bp, including barcodes and adapter sequences) and purified by using Diffinity RapidTip2 PCR purification tips (Diffinity Genomics, NY). Amplicons from different samples were pooled at equimolar concentrations and sequenced by using a paired-end Illumina MiSeq 500 cycle kit with 5% PhiX to increase read diversity.

### HOTS, New England, Mo'orea, and Shedd Aquarium water column data.

We analyzed existing 16S rRNA gene data sets to compare the OV community composition to those of surface water from three nearshore ocean environments and another saltwater-aquarium water column. The first data set comprised 27 samples over 2 years from a depth of 25 m at Station ALOHA (HOTS) and was downloaded from the NCBI Sequence Read Archive (SRA) database (accession number SRX556089). The second and third data sets were available in-house. The second set comprised surface water samples collected from a site offshore from Rhode Island (here “New England”) at four seasonal time points (Fall, Winter, Spring, and Summer [two replicate samples per time point, for eight samples total]) (18). The third data set comprised surface water samples from nearshore waters surrounding tropical coral reefs around Mo'orea, French Polynesia (56). The final data set was obtained from the Shedd Aquarium in Chicago (B. Van Bonn, personal communication) and comprised 50 samples from a 1,600-gallon artificial seawater aquarium water column before, during, and after a large water change (11).

### Metagenome (DNA) sequencing.

To explore the functional roles of dominant OV microbes, we sequenced the metagenomes of one bloom sample and one nonbloom sample, from 5 August 2016 (bloom) (sample 0805) and 28 September 2016 (nonbloom) (sample 0928) (see Table S2 in the supplemental material). For the metagenomes, DNA was extracted from one of the replicate Sterivex cartridges from each date, as described above, for amplicon sequencing. DNA was processed by using the Nextera XT DNA sample prep kit and sequenced by using a paired-end Illumina MiSeq 600 cycle kit.

### RNA extraction and metatranscriptome (cDNA) sequencing.

One of the four replicate Sterivex cartridges from each of the two samples used for metagenome analyses (samples 0805 and 0928) was also processed for metatranscriptome sequencing. RNA was extracted by using a modification of the mirVana microRNA (miRNA) isolation kit (Ambion). Filter cartridges were thawed on ice, RNA-stabilizing buffer was then expelled and discarded, and cells were lysed by the addition of lysis buffer and miRNA homogenate additive (Ambion) directly to the cartridges. Following vortexing and incubation on ice, lysates were transferred to RNase-free tubes and processed via acid-phenol-chloroform extraction according to the kit protocol. The Turbo DNA-free kit (Ambion) was used to remove DNA, and the extract was purified by using the RNeasy MinElute cleanup kit (Qiagen). The Ribo-Zero rRNA removal kit for bacteria (Epicenter) was used to deplete rRNA sequences, and cDNA was generated by using the ScriptSeq v2 transcriptome sequencing (RNA-Seq) library preparation kit (Epicenter) and sequenced on an Illumina MiSeq instrument by using a paired-end Illumina MiSeq 600 cycle kit.

### 16S rRNA gene amplicon data analysis.

Raw paired-end Illumina reads from the OV, New England, Mo'orea, and Shedd Aquarium data sets were trimmed with Trimmomatic-0.36 (38), using the default leading and trailing parameters, a quality control sliding window at 4 bases with a minimum *q* score cutoff of 25, and a minimum length cutoff of 150 bases. The HOTS data were downloaded from the SRA as quality-filtered reads. Forward-paired reads from all data sets were merged into one fasta file and run through Deblur (39) (cutoff length of 150 bp) and QIIME v1.9.1 (40) to assess community composition. The use of Deblur meant that sequences were not clustered into operational taxonomic units but rather that each unique read was treated as a distinct taxon, here referred to as an amplicon sequence variant (ASV). Taxonomy was assigned by using the SILVA 128 database (99% identity level), using BLAST. Sequences classified as chloroplasts were removed from the final table, and samples were rarefied to a depth of 3,748 and aligned with Pynast (41). Uninformative base positions based on the default lane mask were removed, and this alignment was used to generate a phylogeny by using the FastTree algorithm (42). Alpha and beta diversity metrics, including weighted and unweighted UniFrac and principal-components analyses, were generated by using the QIIME script core_diversity_analysis.py. OV samples were categorized numerically by time period, as follows: 1 for 18 May to 12 July 2016, 2 for 5 August to 29 August 2016, 3 for 28 September to 15 December 2016, 4 for 29 December 2016 to 13 April 2017, and 5 for 27 April to 29 July 2017.

### Metagenome analysis.

Raw reads were quality trimmed as described above. For taxonomic breakdown of the metagenome, reads were merged by using FLASH v. 1.2.9 (43), and the resulting fastq files were submitted to the NCBI Sequence Read Archive. Ribosomal reads were removed from the merged reads by using RiboPicker (44). Non-rRNA reads were queried against the NCBI nr database with DIAMOND (45), using BLASTX and a minimum bit score of 50. The output was imported into MEGAN Community Edition version 6.8.20 (46) for taxonomic assignment. To compare relevant genes and taxa between time points, the sample 0928 transcriptome was randomly subsampled to the same number of reads as that for sample 0805 (8,963,441 reads).

Two coassemblies were generated from the quality-trimmed metagenomic reads of both samples (samples 0805 and 0928) by using Megahit v.1.0 (47), with the default parameters for paired-end assembly. One coassembly was generated with a minimum contig size of 2,500 bp (for metagenome analysis), and the other was generated with a minimum contig size of 1,000 bp (for metatranscriptome analysis [see below]). Assembly statistics were calculated by using MetaQUAST (48) and are presented in Table S3 in the supplemental material. Short reads for each sample were mapped back to the coassemblies by using Bowtie2 (49), and the mapping percentages are given in Table S4. Genomic bins, referred to here as metagenome-assembled genomes (MAGs), were then generated by using the reads from each sample and the coassembled metagenome (2,500-bp assembly), using MaxBin 2.0 (50). The recruitment of short reads to each MAG was calculated by using Bowtie2. Metagenomes were imported into anvi'o v2.4.0 (51) for alignment, visualization, and manual refinement, including the removal of contigs containing eukaryotic rRNAs and plasmids. Refined MAGs were analyzed for completeness and contamination by using CheckM (52). Binning results are presented in Table S5, and bin read recruitment percentages for each sample are presented in Table S6. Open reading frames were identified by using Prodigal (48) and annotated by using Prokka (53). MAG contigs were also queried against the NCBI nr database by using BLASTX. Prokka annotations were queried for genes involved in aerobic and anaerobic respiration, photosynthesis, and the oxidative stress response, and the resulting hits visualized to determine the contig position and transcription levels in anvi'o. The hits were also confirmed by their presence in the BLAST results. Putative plasmids were identified in anvi'o by the manual screening of gene annotations on contigs with aberrant GC contents and coverage values relative to the MAG average in anvi'o (Fig. S1). Metagenomes were visualized together, including MAGs and rRNA annotations, by using anvi'o.

### Metatranscriptome analysis.

Raw reads were quality trimmed, merged, and processed with RiboPicker, as described above. Quality-filtered, merged reads were submitted to the NCBI SRA. Non-rRNA reads were queried against the NCBI nr database with DIAMOND by using BLASTX and a minimum bit score of 50, and the output was imported into MEGAN Community Edition version 6.8.20 (46) for taxonomic assignment, as described above for the metagenomes. To compare relevant genes and taxa between time points, the sample 0928 transcriptome was randomly subsampled to the same number of reads as that of sample 0805 (3,340,400 reads) (see Table S4 in the supplemental material). The DIAMOND results were used to count transcripts encoding cyanophycin synthetase and cyanophycinase genes as well as those associated with the alphaproteobacterial lineage and the genus Sulfurimonas.

Non-rRNA reads were then mapped to both metagenome coassemblies (2,500 bp and 1,000 bp) by using Bowtie2 (49). Reads mapped to the 1,000-bp assembly were used for downstream analyses due to a higher recruitment level than that of the 2,500-bp assembly (Table S4). Mapped reads were imported into anvi'o v2.4.0 for visualization.

### Phylogenies: MAGs and cyanophycinase genes.

A phylogeny was generated to assess relatedness among three near-complete MAGs (MAG 1 from sample 0805 [here 0805_001] and MAGs 1 and 2 from sample 0928 [here 0928_001 and 0928_002]) and several marine alphaproteobacterial genomes available from the NCBI (see Table S7 in the supplemental material). CheckM (52) was used to generate a concatenated alignment of 43 single-copy marker genes from all MAGs and reference genomes. Best-model-fit estimation and maximum likelihood tree generation were performed on the amino acid alignment with MEGA 7.0.21 (54), using the L+G+I+F model with 999 bootstraps.

Genes annotated as cyanophycin synthetase and cyanophycinase by both Prokka and BLAST for each of the three MAGs were aligned by using Muscle and placed in a phylogeny with all available full-length genes with the same annotation in the NCBI database by using MEGA. Phylogenies were run by using the L+G+I+F model with 999 bootstraps.

### Accession number(s).

All OV data were submitted to the NCBI Sequence Read Archive under accession number SRP126657 (BioProject number PRJNA417313).

## Supplementary Material

Supplemental material
