# Supplementary material for: Microbiome Dynamics in a Large Artificial Seawater Aquarium
Source: Appl Environ Microbiol. 2018 May 1;84(10):e00179-18. doi: 10.1128/AEM.00179-18 (PMC5930379; doi:10.1128/AEM.00179-18)
Supplement: Supplemental material [file supp_84_10_e00179-18__index.html]

Supplemental material 

# Microbiome Dynamics in a Large Artificial Seawater Aquarium

## Supplemental material

- Supplemental file 1 -

  Subset of the metagenome and metatranscriptome visualization provided in Fig. 5 (Fig. S1); rarefaction curves (Fig. S2); average relative abundances of major microbial phyla (Fig. S3); correlation between the relative abundances of the families *Rhodobacteraceae* and *Kordiimonadaceae* in 16S rRNA gene amplicon datasets (Fig. S4).

  PDF, 1.5M
- Supplemental file 2 -

  Chemical parameters (Table S1); sampling schedule data (Table S2); assembly statistics for the two metagenomic coassemblies (Table S3); sample statistics (Table S4); CheckM genome completeness statistics (Table S5); unmapped and mapped metagenome/metatranscriptome reads (Table S6); reference genomes used to build the phylogeny with the three MAGs (Table S7); rRNA annotations (Table S8); genes related to aerobic respiration (Table S9) and oxidative stress tolerance (Table S10) and the MAGs in which they were identified; putative plasmids and ORF annotations (Table S11); transcription levels of cyanophycin-related genes (Table S12).

  XLSX, 54K
