# Supplementary material for: Microbiome Dynamics in a Large Artificial Seawater Aquarium
Source: Appl Environ Microbiol. 2018 May 1;84(10):e00179-18. doi: 10.1128/AEM.00179-18 (PMC5930379; doi:10.1128/AEM.00179-18)

Figure S1: A subset of the metagenome and metatranscriptome visualization provided in Figure 5, highlighting the contigs annotated with rRNA sequences and putative plasmids. Contigs that clustered by tetranucleotide frequency with one of the MAGs but featured different GC content and coverage values were identified as putative plasmids.

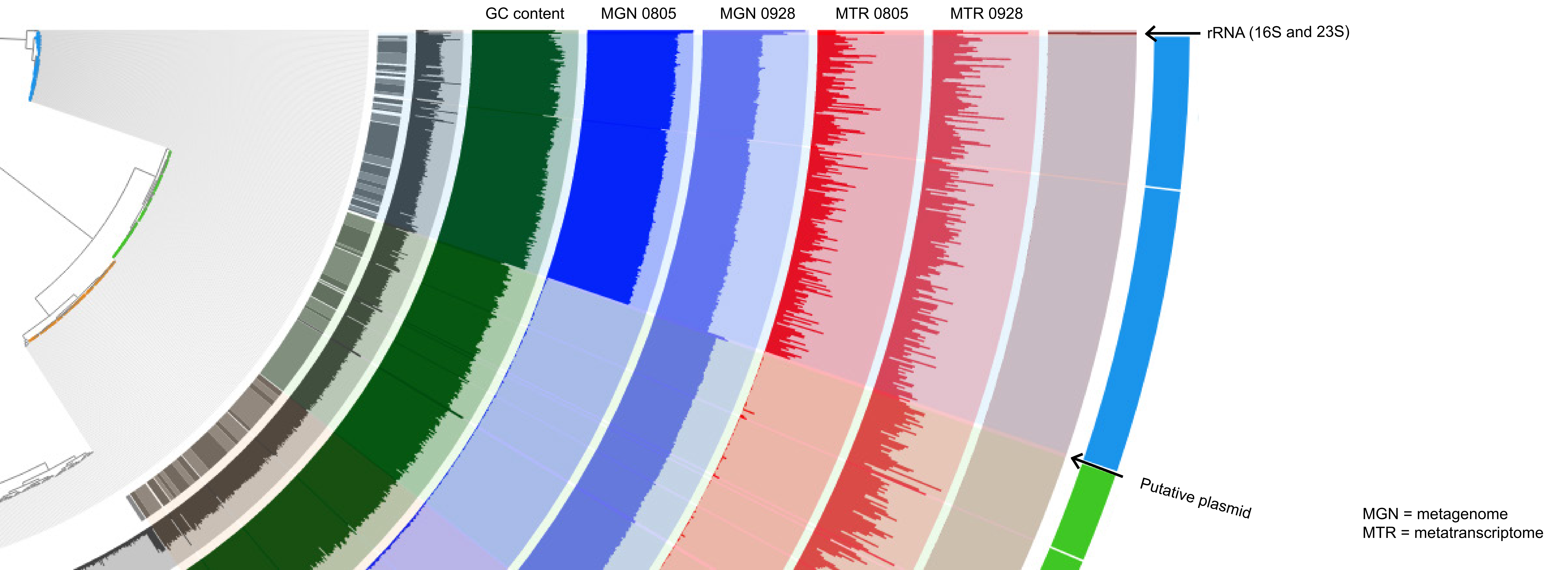

Figure S2: Rarefaction curves from two aquariums (the Ocean Voyager and Shedd Aquarium) and three natural marine surface water column communities (Station ALOHA in Hawai'i, French Polynesia, and New England). Each data set was rarefied to 3748 sequences.

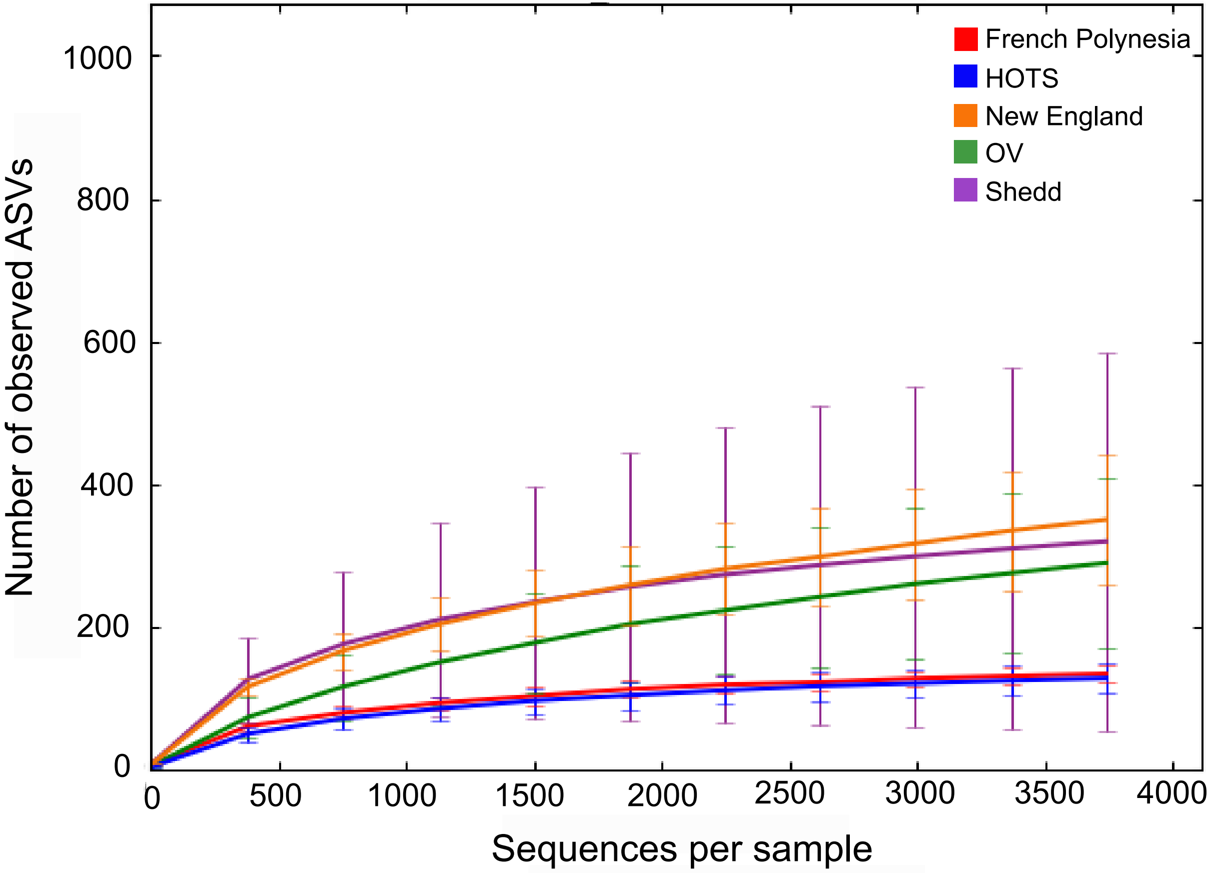

Figure S3: Average relative abundances of major microbial phyla in the OV and HOTS samples.

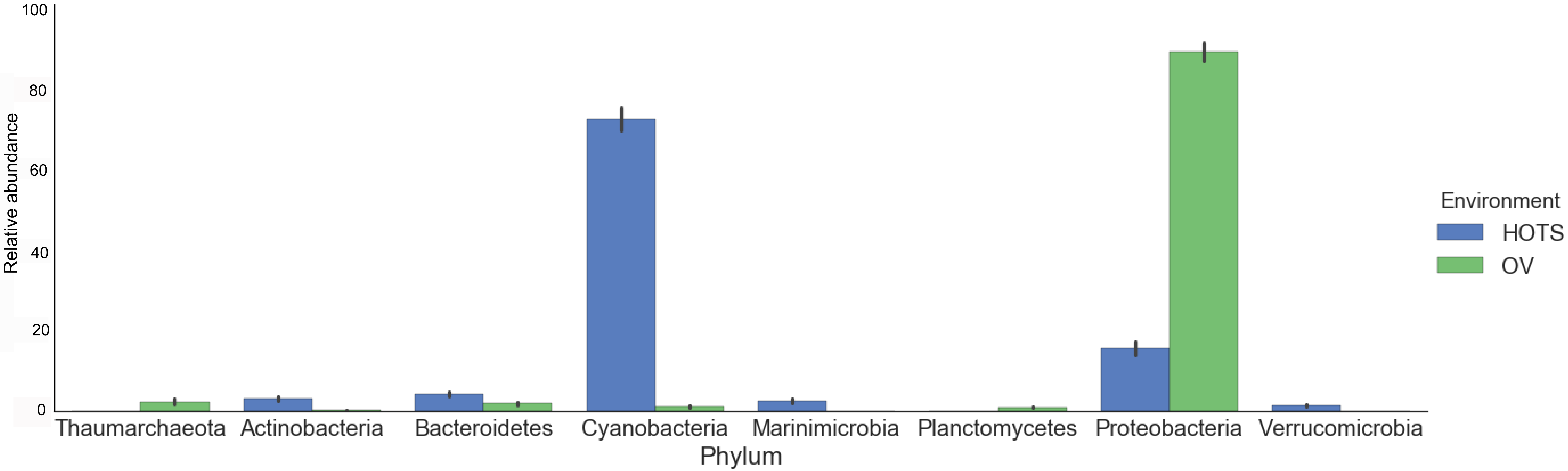

Figure S4: : Correlation between the relative abundances of the families Rhodobacteraceae and Kordiimonadaceae in 16S rRNA gene amplicon datasets. Samples with zero counts of Kordiimonadaceae are removed from this analysis.

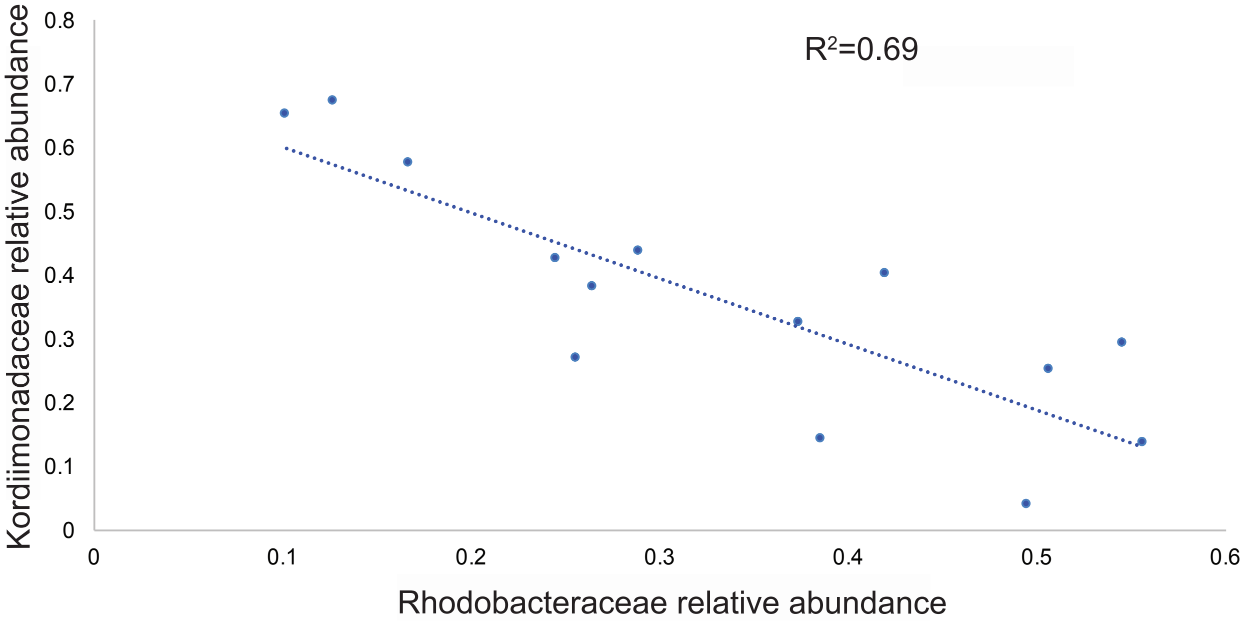

Supplement: Supplemental material [file AEM.00179-18_zam010188504s1.pdf]
